# The Economic Impact of the SARS-COV-2 (COVID-19) Pandemic in Spain

**DOI:** 10.3390/ijerph18094708

**Published:** 2021-04-28

**Authors:** Jaime Pinilla, Patricia Barber, Laura Vallejo-Torres, Silvia Rodríguez-Mireles, Beatriz G. López-Valcárcel, Luis Serra-Majem

**Affiliations:** 1 Department of Quantitative Methods for Economics and Management, University of Las Palmas de Gran Canaria, 35017 Las Palmas, Spain; patricia.barber@ulpgc.es (P.B.); laura.vallejo@ulpgc.es (L.V.-T.); beatriz.lopezvalcarcel@ulpgc.es (B.G.L.-V.); 2 Canary Health Service, Hospital Universitario de Gran Canaria Dr. Negrín, 35010 Las Palmas, Spain; silvia.rodriguezmireles@ulpgc.es; 3 Research Institute of Biomedical and Health Sciences (IUIBS), University of Las Palmas de Gran Canaria, 35016 Las Palmas, Spain; lluis.serra@ulpgc.es

**Keywords:** economic impact, uncertainty, COVID-19, Bayesian structural time series, Spain

## Abstract

Background: The COVID-19 pandemic has hit both the Spanish economy and the population’s health hard. The result is an unprecedented economic and social crisis due to uncertainty about the remedy and the socioeconomic effects on people’s lives. Methods: We performed a retrospective analysis of the macroeconomic impact of the COVID-19 pandemic in 2020 using key indicators of the Spanish economy for the 17 Autonomous Communities (ACs) of the country. National statistics were examined in the search for impacts or anomalies occurring since the beginning of the pandemic. To estimate the strength of the impact on each of the indicators analyzed, we used Bayesian structural time series. We also calculated the correlation between the rate of GDP decline during 2020 and the cumulative incidence of COVID-19 cases per 100,000 inhabitants in the ACs. Results: In 2020, the cumulative impact on the gross domestic product was of −11.41% (95% credible interval: −13.46; −9.29). The indicator for business turnover changed by −9.37% (−12.71; −6.07). The Spanish employment market was strongly affected; our estimates showed a cumulative increase of 11.9% (4.27; 19.45) in the rate of unemployment during 2020. The worst indicators were recorded in the ACs most economically dependent on the services sector. There was no statistical association between the incidence of COVID-19 in 2020 and the fall in GDP in the ACs. Conclusions: Our estimates portray a dramatic situation in Spain, where the COVID-19 crisis has had more serious economic and health consequences than in other European countries. The productive system in Spain is too dependent on sectors vulnerable to the pandemic, and it is necessary to design and implement profound changes through the European Next Generation program.

## 1. Introduction

On 5 January 2020, the World Health Organization (WHO) published its first technical report about a new coronavirus, the SARS-CoV-2 [1]. Twenty-five days later, the WHO declared that the pandemic, called COVID-19, that had been caused by this coronavirus constituted a public health emergency of international importance [2]. We prepared this article one year after the official recognition of the existence of the SARS-CoV-2, and the progression of the COVID-19 pandemic still seems endless. New contagions and losses of human life are still continuing day after day.

**Figure 1:**
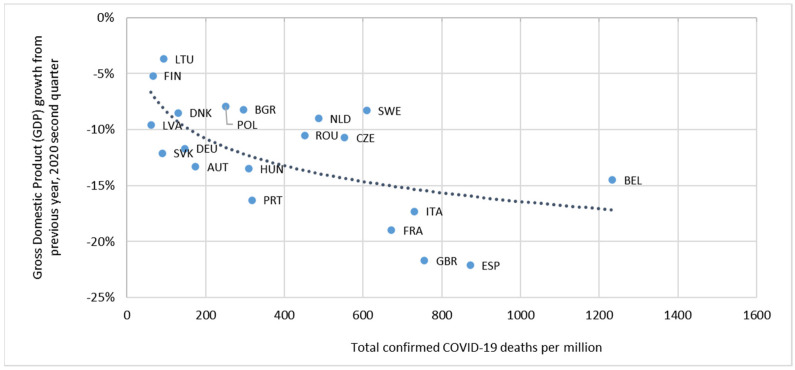
Impact of the COVID-19 pandemic on health and economic growth in European countries. Source: Own preparation, based on OECD statistics and John Hopkins University CSSE COVID-19 data.

Spain is a very interesting case study because it is the European country with the largest drop in gross domestic product (GDP) in 2020 [3] and with the highest rates of COVID-19 incidence, hospital admissions, and deaths in 2020, especially during the first wave [4], with an excess mortality over the expected 61% between March and May [5]. Doubts about the management of the pandemic led a group of scientists to publish a letter in the Lancet in August calling for an audit of Spain [6]. Spain appears among the group of countries in the European Union with the highest COVID-19 death rate and it is also showing the worst economic indicators. The enormous differences among countries suggest that there is a relationship between the health crisis and the economic impact, see Figure 1.

In Spain, the COVID-19 pandemic is having a severe impact on the national health system that was not prepared for it due to insufficient financing following the cutbacks of the previous economic crisis [7] and due to serious structural imbalances [8]. In 2018, health expenditure in Spain was about 2445 euros per capita, adjusted for purchasing power; it was 12% lower than in the European Union (average) [9].

There is huge heterogeneity among countries in the management of the pandemic. The COVID-19 health system response monitor [10] is a platform created by the WHO Regional Office for Europe, the European Commission, and the European Observatory on Health Systems and Policies for tracking the responses of the countries to the COVID-19 crisis. In Spain, the main strategies used against the successive epidemic waves of COVID-19 were a strict lockdown and home confinement for three months starting 14 March 2020, and then social distancing, compulsory use of masks, restrictions on movement, teleworking, and restrictions and barriers in many economic sectors, especially hotels and catering.

Successive rebounds in COVID-19 incidence have resulted in a worrying deceleration in many sectors of the Spanish economy, and a huge increase in economic agent’s uncertainty about the future. It is unknown how serious the next wave of infections will be or how quickly the vaccinations or other treatments will be successful. In this atmosphere of uncertainty, aggregate demand and demand for investment are declining drastically [11]. Moreover, the spread of the consequences of the pandemic to countries throughout the world is resulting in restrictions on movements between countries, with a direct effect on tourism, which is the main driver of the Spanish economy.

Three mechanisms for the economic impact of the pandemic have been considered: a direct mechanism via demand and consumption reduction (due to uncertainty, households’ change consumption with savings); indirect impact via the financial markets (drop of the value of assets), and fall in the supply of, and demand for, work and employment, which ultimately results in losses of jobs and reduced activity [12]. The disease has a direct effect on the economy, and in turn poverty, unemployment, and increase in economic inequalities may deteriorate health in the medium and long term [13,14].

Conceptually, the decline in GDP and other economic indicators attributable to the pandemic can be divided into three components, the policy implications of which are quite different: (1) Those that are exogenous to the country and out of the control by the government as they depend on the dynamics of the pandemic globally and on the countries that are economically most interconnected with the one being analyzed; (2) Those that are due to the necessary restrictions on social interactions and economic activity. They are determined by the pandemic; therefore, they are also somewhat exogenous; (3) Those that cause a fall in the GDP due to “avoidable” restrictions on economic activity that go beyond the optimal restrictions.

There is rich economic literature on the optimal restrictions [15]. There is a debate on the optimal strategy to control the pandemic and minimize its economic and health effects, with two main approaches, the so called “red zone strategy” and the “green zone strategy”. The latter, followed by countries such as Australia, New Zeeland, and Taiwan, started with strict lockdowns until the number of cases was consistently zero, and then reopened all the normal economic activities, with a close control of people coming in from red zones abroad [16]. The two strategies manage differently the trade-off between intensity and duration of the constraints to the economic activity.

In a previous article [17], we estimated the economic loss in GDP in different countries attributable to the COVID-19 pandemic in 2020 and 2021 with a simple method consisting in comparing the forecasts released by the IMF after the pandemic and before its aftermath (the counterfactual). In this paper, we use a more sophisticated method, Bayesian structural time series models, to measure the economic impact of the COVID-19 pandemic for Spain in 2020, and analyze the differences between ACs, which are responsible for the management of the health crisis. We estimate the impact attributable to the epidemic on the main macroeconomic aggregate (GDP) and on short-term indicators of production and supply, demand, and unemployment. We explore the possible association between the economic impact on GDP and the impact on health (cumulative incidence rate in 2020) with data from the CAs.

## 2. Materials and Methods

### 2.1. Data

We analyzed the trend in indicators of the macroeconomic aggregates, gross domestic product; production, supply, and demand; and labor market, by Autonomous Community (AC). Indicators used were quarterly or monthly time series.

The data were compiled by the National Statistics Institute (INE in Spanish), the Independent Authority for Fiscal Responsibility (AIReF in Spanish), the Centre for Sociological Research (CIS in Spanish), and the Ministry of Social Security.

The indicators selected were as follows:

I. General macroeconomic aggregate.
  - Gross domestic product volume index (GDP). Quarterly data by ACs, seasonally and calendar adjusted. Time series from the first quarter of 2000 to the fourth quarter of 2020. GDP is the most frequently used measure for the overall size of an economy. GDP-index in volume represents the variations in the volume of production of an economy.
II. Production, supply, and demand.
  - Industrial production index (IPI). Monthly data by ACs (base 2015). Time series from January 2002 to December 2020. The (IPI) measures the monthly evolution of the productive activity of the industrial branches, that is, of the extractive, manufacturing, production, and distribution activities of electrical energy, water, and gas.
  - Business turnover index (BTI). Monthly data by ACs (base 2015). Time series from January 2005 to December 2020. The BTI is an indicator that measures the short-term evolution of turnover, as a whole, for non-financial economic sectors.
  - Consumer confidence index (CCI). Monthly data at the national level. Time series from September 2004 to December 2020. The CCI offers an insight into consumers’ spending intentions, asking them about their current perception of the country’s economy and its prospects, their family economy, and employment.
III. Labor Market.
  - Unemployment rate, percentage of unemployed workers in the total labor force. Quarterly data by ACs and economic sector (agriculture, industry, and services). Data from the Economically Active Population survey (EPA in Spanish). Time series from the first quarter of 2008 to the fourth quarter of 2020.

The above-mentioned indicators are part of the Spanish national accounting system, their purpose being to reflect, in a composite and quantitative manner, the main features of economic activity in a specific period: nationally, regionally, and by sector [18].

### 2.2. Statistical Analysis

To estimate the economic impact of the COVID-19 pandemic, we performed a modelling, by means of the Bayesian structural time series (BSTS) [19,20], of each of the indicators using the information available in each indicator during the period before the beginning of the state of alert in Spain in March 2020. The BSTS models provide a flexible analytical framework to decompose the components of the time series, incorporating the prior information, and capturing the evolving nature of model parameters. The BSTS approach incorporates a time series component, including local linear trends and seasonality, and a regression component to model both temporal changes as well as impacts of the intervention. The causal impact of the intervention is calculated as the difference between the observed value of the time series and the (unobserved) value that would have been obtained under an alternative circumstance (without the COVID-19 pandemic). The BSTS models are applied across the board in all scientific fields. In the past few years, the use of BSTS in the evaluation of health policy interventions has become more widespread [21,22,23]. At present, there already exist BSTS applications to address the COVID-19 effects [24].

In our BSTS model, the dynamics of the variable of interest are explained by starting with the equation.

(1) $$y_{t}=\mu_{t}+\tau_{t}+\varepsilon_{t}$$

The first and second state components,$\;\mu_{t}$ and $\tau_{t}$, are the trend at time *t* and the seasonal component, respectively. Our model assumes a local linear trend in which the expected increase in μ between *t* and *t* + 1(δ) presents a random walk pattern.

(2) $$\mu_{t}=\mu_{t-1}+\delta_{t}+\upsilon_{t}$$

(3) $$\delta_{t}=\delta_{t-1}+\nu_{t}$$

The seasonal component is represented by the state component $\tau_{t}$, which can be interpreted via a set of 12 dummy variables (4 dummy variables in quarterly time series) with dynamic coefficients constrained to have zero expectation over a year.

(4) $$\tau_{t}=-\sum_{s=1}^{12-1}\tau_{t-s}+w_{t}$$

The error terms $\varepsilon_{t}$ and $\eta_{t}=\left(\upsilon_{t},\;\nu_{t},\;w_{t}\right)$ follow independent Gaussian random noises, N(0, $\sigma_{*}^{2}$). The model is estimated in a Bayesian framework, specifying the prior distributions of the unknown parameters $\theta :\left\{\sigma_{\varepsilon }^{2},\sigma_{\upsilon }^{2},\sigma_{\nu }^{2},\sigma_{w}^{2}\right\}$. The Gibbs sampling is used to simulate the parameters of the model and the posterior predictive distribution over the counterfactual time series, given the observed pre–pandemic activity. Once converged, each Gibbs sampling trajectory may be iterated forward using the estimated state variables and parameters to construct the counterfactual time series.

The actual response is compared with the counterfactual time series. Subtracting this counterfactual time series from the observed response during the pandemic period yields a semiparametric Bayesian posterior distribution for the impact effect. Finally, we can use the samples from the posterior distribution to report the relative cumulative effect caused by the pandemic, including the Bayesian credible interval (CI). We implemented our analysis in R using the Causal Impact package [25,26].

## 3. Results

### 3.1. Gross Domestic Product

**Figure 2:**
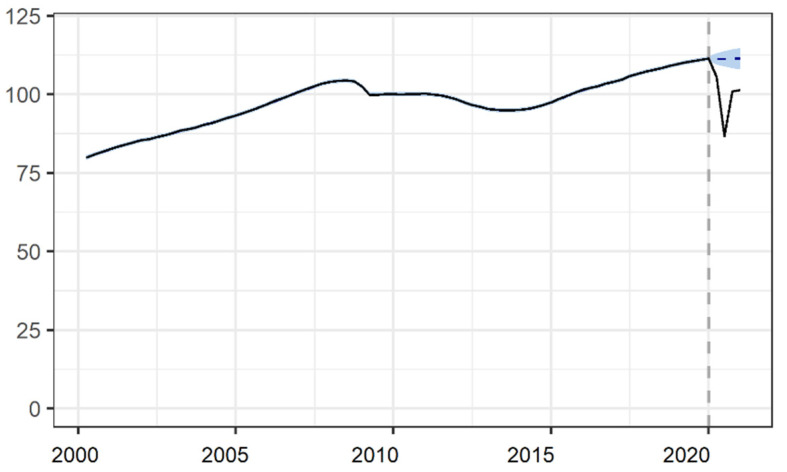
Impact of the COVID-19 crisis on the gross domestic product volume index in Spain.

Figure 2 shows the progression of the time series, with quarterly frequency, of the GDP volume index for the national total. A sharp fall in this index was clearly noticeable from the beginning of the pandemic, in the first quarter of 2020. We estimated a cumulative relative impact of the pandemic on the GDP volume index of −11.41% in 2020 (95% credible interval: −13.46; −9.29), see Table 1. By ACs, those most economically dependent on the services sector, and especially on tourism, were those which showed the greatest negative impact in GDP. This was the case of the Balearic Islands (−19.61% (−21.65; −17.53)) and the Canary Islands (−14.09% (−16.13; −12.03)). At the other end of the scale, the ACs recording the lowest fall in GDP were those in which the agrarian sector has the greatest weight, namely Extremadura (−7.45% (−9.46; −5.42)) and Murcia (−8.42% (−10.92; −5.94)).

### 3.2. Production, Supply, and Demand Short-Term Indicators

**Figure 3:**
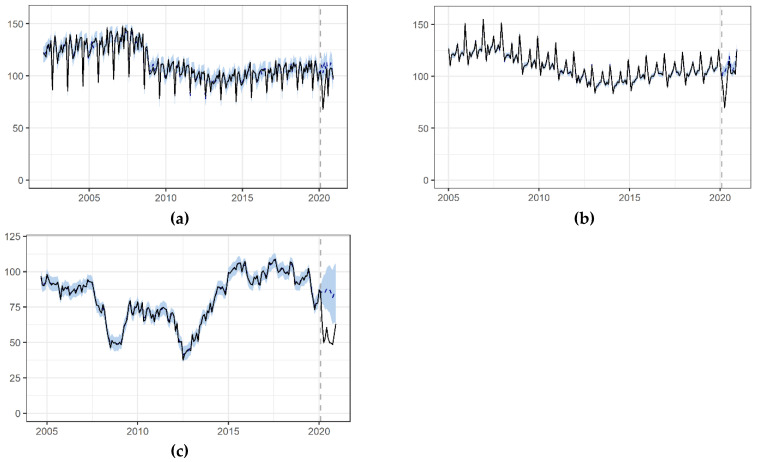
Impact of the COVID-19 crisis on supply and demand in Spain. (**a**) Industrial production index (**b**) Business turnover index (**c**) Consumer confidence index. Frequency: Monthly.

The COVID-19 crisis has impacted the country’s industrial activity, causing a sharp fall in the IPI (see Figure 3a). Nationally, the change was −9.92% (−15.68; −4.26). The two CAs with highest share of the agricultural sector in the GDP on the supply side, Extremadura and Murcia, did not show significant changes in their IPI. The greatest decrease was estimated for the Balearic Islands (−22.36% (−33.14; −11.71)), the Basque Country (−15.65% (−23.01; −8.51)), and Asturias (−15.30% (−21.52; −9.04)).

The reduction in business activity is directly related to the strong fall in demand in Spain during the pandemic, in particular during the strict confinement. We estimate that, nationally, the BTI changed by −9.37% (−12.71; −6.07). Once again, the Balearic Islands and the Canary Islands experienced greater impacts: −16.81% (−20.98; −12.73) and −18.43% (−22.23; −14.6), respectively (see Figure 3b and Table 2).

Although the drop in both the business turnover and the IPI may be related to the strong fall in demand in Spain during the pandemic, in particular during the strict lockdown of March–June, a direct short-term indicator of the consumer’s demand is the consumer confidence index. We estimate that, nationally, the CCI changed by −36% (−52%; −21%) (Figure 3c and Table 2).

### 3.3. Labor Market: Unemployment

**Figure 4:**
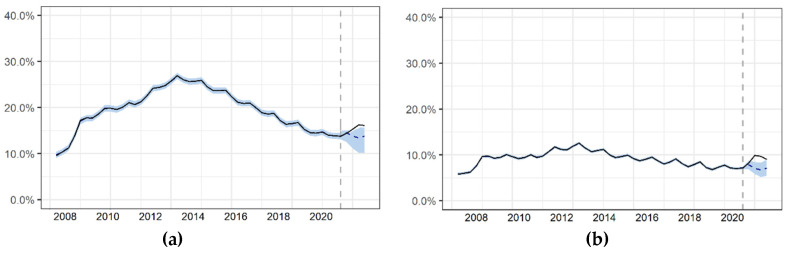
Impact of the COVID-19 crisis on the employment market in Spain. (**a**) Total unemployment rate (**b**) Services sector unemployment rate. Frequency: Quarterly.

The COVID-19 crisis has also strongly affected the Spanish employment market. We estimate that during 2020 there was a cumulative increase of 11.9% (4.27; 19.45) in the unemployment rate. The ACs with the highest growth in the unemployment rate were the Balearic Islands (58.1% (20.91; 95.26)), Madrid (24.2% (8.73; 39.75)), and Catalonia (20.8% (7.49; 34.12)). See Figure 4a and Table 3.

Most of the lost jobs were in the service sectors. Nationally, in 2020 we found a cumulative increase in the unemployment rate in services of 27.68% (9.96; 45.4). The Balearic Islands were the most affected, with a cumulative increase of 74.82% (26.93; 122.7). See Figure 4b and Table 3.

### 3.4. Economics and Health: Is There an Association between Economic Downturn and COVID-19 Incidence?

**Figure 5:**
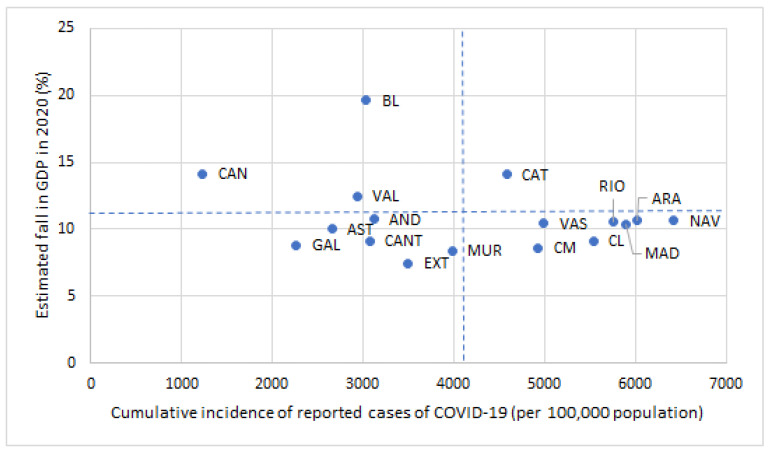
Gross domestic product decrease and COVID-19 incidence by Autonomous Communities. Spain 2020. Source: Own preparation based on Spanish Ministry of Health statistics and our estimates in Table 1.

The linear correlation between the fall in GDP in 2020 and the cumulative incidence of COVID-19 per 100,000 inhabitants in the ACs of Spain is negative, −0.23, apparently showing that the ACs with higher incidence suffered less economically and vice versa, but this correlation is not significant, *p*-value = 0.3688. Three ACs had large falls in GDP with low incidence of the virus (the two islands and Valencia) (Figure 5). Catalonia has suffered more than the average in both economic and health terms. The least affected were the three Autonomous Regions in northern Spain (Galicia, Asturias, and Cantabria) and the most agrarian ACs (Andalusia, Extremadura, and Murcia); these groups, located in the third quadrant of the Figure 5, had proportionally fewer incidences of COVID-19 and a smaller fall in GDP.

## 4. Discussion

The economic impact of a pandemic does not happen gradually, nor is there any date for its end. The effects on economic activity depend on the progression of the pandemic, and in particular on the measures taken to contain it [12]. In Spain, those measures caused an economic slowdown that resulted in a fall in revenue and a sharp increase in unemployment during 2020. The pandemic spread quickly to countries throughout the world and provoked restrictions on movements, with a direct effect on tourism and a reduction in international demand for goods and services.

Changes in the GDP constitute an indicator of obvious importance for measuring economic impacts in any country. We estimate that the adverse impact of the COVID-19 pandemic on GDP in Spain in 2020 was of 11.41% (Table 1). Our estimates are similar to those of BBVA Research [27].

The strict confinement measures between March and May, 2020, resulted in an immediate decrease in demand, as they restricted access to the majority of the establishments selling goods and services, and also because families, faced with such a dismal outlook, reduced their consumption and began to save more. This mechanism, which acts through the demand channel, is one of the three that have been described to explain the impact of the pandemic on the GDP [12]. The other two are indirect impacts via the financial markets, and the fall in the supply of, and demand for, work and employment, which ultimately results in losses of jobs and reduced activity [28].

Specialization in the agricultural sector has proven to be, according to this study, a protective factor against the fall in GDP. Only in Extremadura, Murcia, and Castilla-La Mancha, the three ACs where the agricultural sector has a greater relative weight, was the decline in GDP attributable to the pandemic less than 9% according to our estimates. The primary sector provided an essential service in maintaining supplies of food to citizens during the COVID-19 pandemic. This sector has managed to deal with the effects of the pandemic as households replaced consumption outside the home with consumption at home [29]. In contrast with the decline of one of the sectors that the pandemic has hit the hardest, the hotel and catering sector, there has been a significant and sustained growth in general food products, especially fresh products, sold directly to households [28].

At the other end of the scale, the communities whose economies are the most dependent on the services sector, particularly on tourism-related sectors, are the ones that have suffered the biggest impact on their GDP. We estimate changes of −19.61% and −14.09% in GDP in the Balearic Islands and the Canary Islands, respectively (see Table 1). The tourism sector weighs heavily in the national GDP, and even more intensely in specific ACs such as the two island regions [30]. In Spain, tourism accounts for 11.8% of the GDP and 13.5% of the employment, whereas in the OECD countries, tourism represents, on average, 4.4% of the GDP and is connected with 6.9% of the jobs [31].

No tourists arrived in our country in April or May, and it was not until June, with the partial lifting of the restrictions on movements, that the first post-pandemic tourists were received. The summer season closed with a cumulative fall of 87.11% in the number of tourists [32].

The COVID-19 crisis has caused an unprecedented fall in the IPI; according to our estimates, the change was −9.92%. During the period of strict confinement, the manufacture of durable consumer goods and capital equipment was affected the most, with respective falls of 69.0% and 57.8% compared with last year’s figures [33]. Apart from construction, the most important industrial sub-sectors in Spain are the automobile industry and the food and beverage industry. The former is of crucial importance because of both the internal demand and the volumes exported. According to data from the manufacturers’ association, compared with 2019, the production of vehicles in Spain declined by 19.6% in 2020 [34]. With regard to production in the food industry, though it is partially for consumption by the resident population (in the home and outside it), it is mainly for tourists and for export.

Nationally and between March and December, 2020, the cumulative number of companies registered as employers with the Social Security authorities decreased. For many companies, the fall in demand resulted in asphyxiation, obliging them either to reduce their supply or to cease trading permanently. Most of the business closures occurred at the beginning of the confinement, in March [35].

Companies whose employees are only temporarily laid off (in Spanish, by an ERTE: temporary employment regulation) are excluded from the registration in Social Security statistics. In the northern industrial regions of Navarre and the Basque Country, ERTE has been used massively as an instrument, leading to fewer permanent layoffs. At the opposite extreme, the Balearic Islands and the Canary Islands had the worst of it, because some companies related to tourism closed permanently during the first months of the pandemic.

Possibly, default on payments and drop in domestic demand were important causes of the decline in production and turnover of the Spanish industry during the 2020 COVID-19 crisis. Our results show that the BTI (national) changed by −9.37% (−12.71; −6.07). Private consumption in Spain represents approximately 60% of GDP, so it is directly responsible for a large proportion of the changes in the economy [36]. In situations like the current one, in which foreign trade can take a long time to recover, it is internal demand that must try to redress the slowdown in the economy. Measures such as the ERTEs are trying to soften the decline in families’ incomes. However, the effect of these policies continues to be weakened by great uncertainty about the future and low confidence in the performance of the economy. Eurobarometer data show that in January 2021 more than 50% of Spaniards have pessimistic expectations about the economy and employment [37]. Our results show that the CCI (national) changed by −36% (−52; −21).

The characteristics of the Spanish employment market—poor skills, a high proportion of temporary jobs, high unemployment, and low salaries (especially in the services sector and among young people)—are the clearest explanation for the vulnerability and inequality that exist in Spain. Our estimates show a cumulative increase of 11.9% (4.27; 19.45) in the national unemployment rate during 2020. It should be noted that the reduction in employment is greater than that shown by these figures because the workers under ERTEs are still regarded as employed people by the Active Population survey.

The impact of the COVID-19 pandemic on the employment market has not been homogeneous throughout the Spanish provinces. Those with a less diversified industry and a greater presence in the service sectors are suffering the most. In short, our calculations of the impact of the COVID-19 pandemic in 2020 reveal a dramatic situation for Spain, for two interconnected reasons: (1) the greater impact of the epidemic (incidence and morbi-mortality) [4] and (2) the fragility of a productive system that had to confront the changes induced by the pandemic that include more prospects of teleworking, increased foreign dependence, dependence on income from tourism, and less development of technology.

This study had some strengths and limitations. It used robust statistical Bayesian models for estimating the impact attributable to the COVID-19 pandemic for a selection of short-term indicators. The BSTS models constitute an advance with respect to the traditional intervention analysis in time series using static regression models or ARIMA models. The static regression models assume that pattern of a time series is steady over time, which is seldom true. On the other hand, the forecasts under ARIMA models depend on the previous patterns of the series along with preceding forecasting errors. The forecasts from BSTS models are less dependent on hypothesized specifications. However, the analytical computation of the Bayesian posterior distribution is extremely complex. The numerical computations are carried out using Markov Chain Monte Carlo methods with a high computational cost.

In regard to the study limitations, in times of uncertainly, the credibility intervals are wide, and consequently we are not able to give more accurate estimates. As for the selection of indicators, although we also considered using the number of companies registered with the Social Security as an indicator, it had shortcomings, which led us not to use it. During the COVID-19 crisis, many companies were forced to stop working. As of March 2020, many of them have been deregistered from Social Security. However, as the ERTES keeps the employment situation of most workers frozen, the number of companies deregistering from the SS is a downward biased indicator of the destruction of the productive sector.

From a methodological perspective, there is no “golden” method to assess the COVID-19 pandemic’s causal effect on the Spanish economy. Approaches using longitudinal data, including the BSTS models, cannot capture the actual impact of the pandemic, just an approximation of it. Nevertheless, other employed approaches use a country or some countries clustered as a control group, but they also present methodological constraints. The economic impact estimation of the COVID-19 crisis is a demanding task because of the complex interdependencies between services and products belonging to different countries and economic sectors. From our point of view, the fact that all ACs are directly exposed to the effects of the COVID-19 crisis makes it impossible to find a control group to be used to build a counterfactual non-COVID-19 pandemic scenario.

## 5. Conclusions

In this article we estimate the economic impact attributable to the epidemic in 2020 in Spain, but its consequences are still unfolding. The economic crisis due to the COVID-19 pandemic is dragging on long enough to have lasting effects, and no one knows for sure when it will end.

The causal relationships between COVID-19 incidence and economic problems, both in terms of GDP and its distribution, are complex. We analyzed the case of Spain as a paramount example of serious impacts both in the population health and in the economy.

From a policy standpoint, the most relevant estimates would not be the total loss of economic activity (GDP) attributable to the pandemic, but the portion that is unavoidable even with the best policy interventions. International organizations that monitor and track COVID-19 policies are a valuable source of information for international comparative studies. In the present study we estimate the economic impact of the COVID-19 pandemic in Spain during 2020 with some disaggregation by economic sectors and ACs. The main result is that there is great heterogeneity between Autonomous Communities, with those most dependent on tourism being the most negatively affected and, at the opposite pole, those with a relative specialization in the primary sector being the least affected. We did not find a significant correlation between the economic fall in GDP in 2020 attributable to the COVID-19 pandemic and the cumulative incidence of infections.

A better allocation of resources is needed to shorten the recovery period and stimulate economic growth. One of the key factors to recovery of the economy is to diversify it with vigorous investments in promising sectors, promoting digitalization, the use of clean energies, and R&D. The availability of EU funds can be a great opportunity to diversify the Spanish economy and, at the same time, prepare for future health threats. Better cooperation between administrations is needed at three levels: national, regional, and local. Never have economic policies concentrated so much on health, and never have health policies had such a strong economic impact as during this pandemic. The challenge now is to do some “fine tuning”, lifting or imposing restrictions on the economy, and succeeding at the same time in keeping the number of contagions below the level that would be critical for our medical resources.

## Figures and Tables

**Table 1:** Bayesian structural time series model results for gross domestic product volume index: relative cumulative effect (95% Bayesian credible interval).

Autonomous Community	GDP Volume Index Pandemic Measuring Period (1st Quarter 2020 to 4th Quarter 2020)	
Andalusia	−10.73%	[−12.88; −8.55]
Aragon	−10.71%	[−13.02; −8.36]
Asturias	−10.00%	[−12.22; −7.75]
Balearic Islands	−19.61%	[−21.65; −17.53]
Canary Islands	−14.09%	[−16.13; −12.03]
Cantabria	−9.09%	[−11.09; −7.03]
Castile-La Mancha	−8.56%	[−11.19; −5.93]
Castile and León	−9.12%	[−10.95; −7.28]
Catalonia	−14.07%	[−16.33; −11.82]
Valencian C.	−12.49%	[−14.93; −10.03]
Extremadura	−7.45%	[−9.46; −5.42]
Galicia	−8.81%	[−10.96; −6.62]
La Rioja	−10.61%	[−13.05; −8.16]
Madrid	−10.39%	[−12.51; −8.23]
Murcia	−8.42%	[−10.92; −5.94]
Navarre	−10.64%	[−12.94; −8.34]
Basque Country	−10.43%	[−12.45; −8.38]
Total Spain	−11.41%	[−13.46; −9.29]

**Table 2:** Bayesian structural time series model results for production, supply, and demand: relative cumulative effect (95% Bayesian credible interval).

Autonomous Community	Industrial Production Index Pandemic Measuring Period (March 2020 to December 2020)		Business Turnover Index Pandemic Measuring Period (March 2020 to December 2020)	
Andalusia	−9.63%	[−17.24; −2.02]	−11.39%	[−15.18; −7.71]
Aragon	−10.11%	[−17.52; −2.92]	−8.19%	[−12.01; −4.37]
Asturias	−15.30%	[−21.52; −9.04]	−5.34%	[−8.86; −1.85]
Balearic Islands	−22.36%	[−33.14; −11.71]	−16.81%	[−20.98; −12.73]
Canary Islands	−13.17%	[−18.35; −7.96]	−18.43%	[−22.23; −14.66]
Cantabria	−8.09%	[−14.23; −2.13]	−6.81%	[−10.4; −3.28]
Castile-La Mancha	−8.77%	[−15.14; −2.52]	−5.16%	[−9.14; −1.23]
Castile and León	−9.63%	[−18.00; −1.59]	−8.07%	[−11.42; −4.74]
Catalonia	−10.32%	[−16.24; −4.49]	−11.77%	[−15.41; −8.18]
Valencian C.	−6.76%	[−12.76; −0.82]	−7.86%	[−11.95; −3.8]
Extremadura	−0.35%	[−9.36; 8.81]	−8.08%	[−10.84; −5.38]
Galicia	−12.48%	[−19.52; −5.75]	−3.23%	[−6.74; 0.24]
La Rioja	−13.47%	[−21.05; −6.06]	−6.13%	[−10.17; −2.18]
Madrid	−7.35%	[−13.77; −1.04]	−6.49%	[−10.45; −2.58]
Murcia	−0.31%	[−8.28; 7.53]	−9.61%	[−14.03; −5.17]
Navarre	−14.05%	[−21.31; −7.14]	−6.92%	[−10.19; −3.67]
Basque Country	−15.65%	[−23.01; −8.51]	−5.45%	[−8.86; −2.12]
Total Spain	−9.92%	[−15.68; −4.26]	−9.37%	[−12.71; −6.07]
Consumer Confidence Index Pandemic measuring period (September 2004 to December 2020)		Total Spain −36.26% [−51.82%; −20.84%]		

**Table 3:** Bayesian structural time series model results for the employment market: relative cumulative effect and 95% Bayesian credible interval.

Autonomous Community	Total Unemployment Rates Pandemic Measuring Period (1st Quarter 2020 to 4th Quarter 2020)		Services Sector Unemployment Rates Pandemic Measuring Period (1st Quarter 2020 to 4th Quarter 2020)	
Andalusia	6.7%	[2.43; 11.05]	23.11%	[8.32; 37.89]
Aragon	15.4%	[5.54; 25.25]	34.03%	[12.25; 55.81]
Asturias	4.4%	[1.57; 7.17]	27.52%	[9.91; 45.14]
Balearic Islands	58.1%	[20.91; 95.26]	74.82%	[26.93; 122.7]
Canary Islands	16.8%	[6.06; 27.62]	39.63%	[14.27; 64.99]
Cantabria	10.3%	[3.7; 16.84]	31.73%	[11.42; 52.04]
Castile-La Mancha	5.8%	[2.09; 9.5]	1.60%	[−0.58; 2.62]
Castile and León	6.0%	[2.17; 9.88]	12.72%	[4.58; 20.87]
Catalonia	20.8%	[7.49; 34.12]	44.23%	[15.92; 72.53]
Valencian C.	11.8%	[4.23; 19.27]	19.34%	[6.96; 31.73]
Extremadura	4.9%	[1.76; 8.03]	7.81%	[2.81; 12.8]
Galicia	0.1%	[0.04; 0.18]	7.66%	[2.76; 12.56]
La Rioja	8.5%	[3.06; 13.95]	37.38%	[13.46; 61.31]
Madrid	24.2%	[8.73; 39.75]	41.69%	[15.01; 68.38]
Murcia	3.1%	[1.13; 5.15]	11.26%	[4.05; 18.46]
Navarre	10.0%	[3.59; 16.34]	48.25%	[17.37; 79.12]
Basque Country	4.8%	[1.72; 7.86]	17.54%	[6.32; 28.77]
Total Spain	11.9%	[4.27; 19.45]	27.68%	[9.96; 45.4]

## Data Availability

The data used in this paper were collected from the National Statistics Institute, the Independent Authority for Fiscal Responsibility, and the Ministry of Social Security. The data are available on the respective websites: https://www.airef.es/es/datalab/series-historicas-de-actualizaciones-pib-trimestral-ccaa/ (accessed on 16 January 2021). https://www.ine.es/dyngs/INEbase/es/operacion.htm?c=Estadistica_C&cid=1254736145519&menu=resultados&idp=1254735576715#!tabs-1254736194962 (accessed on 16 January 2021). https://www.ine.es/dyngs/INEbase/es/operacion.htm?c=Estadistica_C&cid=1254736176863&menu=resultados&idp=1254735576778#!tabs-1254736195018 (accessed on 16 January 2021). https://www.ine.es/dyngs/INEbase/es/operacion.htm?c=Estadistica_C&cid=1254736176900&menu=resultados&idp=1254735576799#!tabs-1254736195063 (accessed on 16 January 2021). https://cis.es/cis/opencms/ES/13_Indicadores/Indicadores/ICC/index.jsp (accessed on 16 January 2021). https://www.ine.es/dynt3/inebase/index.htm?padre=979&capsel=979 (accessed on 16 January 2021).

## References

[1] World Health Organization. https://www.who.int/csr/don/05-january-2020-pneumonia-of-unkown-cause-china/es/.

[2] World Health Organization. https://www.who.int/docs/default-source/coronaviruse/situation-reports/20200130-sitrep-10-ncov.pdf?sfvrsn=d0b2e480_2.

[3] International Monetary Fund World Economic Outlook. https://www.imf.org/en/Publications/WEO/Issues/2020/01/20/weo-update-january2020.

[4] Coronavirus Pandemic (COVID-19). https://ourworldindata.org/coronavirus.

[5] Instituto de Salud Carlos III Vigilancia de los Excesos de Mortalidad Por Todas las Causas. https://www.isciii.es/QueHacemos/Servicios/VigilanciaSaludPublicaRENAVE/EnfermedadesTransmisibles/MoMo/Documents/informesMoMo2021/MoMo_Situacion%20a%205%20de%20enero_CNE.pdf.

[6] García-Basteiro A., Alvarez-Dardet C., Arenas A., Bengoa R., Borrell C., Del Val M., Franco M., Gea-Sánchez M., Gestal Otero J.J., López Valcárcel B.G. (2020). The need for an independent evaluation of the COVID-19 response in Spain. Lancet.

[7] López-Valcárcel B.G., Meneu R. (2012). El gasto que está triste y azul: Debe preocupar más la salud que el gasto sanitario. Gac. Sanit..

[8] Sisó-Almirall A. El Desafío de la COVID-19 Para la Atención Primaria y Comunitaria. http://www.aes.es/blog/2020/05/27/el-desafio-de-la-covid-19-para-la-atencion-primaria-y-comunitaria/.

[9] OECD Health at a Glance: Europe 2018. https://www.oecd-ilibrary.org/social-issues-migration-health/health-at-a-glance-europe-2018/health-expenditure-per-capita_health_glance_eur-2018-29-en;jsessionid=85quQOGAsLpny1ZgYaxNpWbC.ip-10-240-5-7.

[10] The Health System Response Monitor (HSRM) Health System Responses to COVID-19. Eurohealth 2020 (26.2). https://eurohealthobservatory.who.int/publications/i/health-system-responses-to-covid-19.

[11] Altig D., Baker S., Barrero J.M., Bloom N., Bunn P., Chen S., Davis S.J., Leather J., Meyer B., Mihaylov E. (2020). Economic uncertainty before and during the COVID-19 pandemic. J. Public Econ..

[12] Carlsson-Szlezak P., Reeves M., Swartz P. (2020). Understanding the Economic Shock of Coronavirus. Harv. Bus. Rev..

[13] Ayala L. (2020). The Potential Consequences of COVID-19 on Poverty.

[14] Alvargonzález P., Pidkuyko M., Villanueva E. (2020). The financial position of the workers most affected by the oandemic: An analysis drawing on the Spanish Survey of Household Finances. Econ. Bull. Banco España Artic..

[15] Brodeur A., Gray D.M., Anik I., Suraiya B. A Literature Review of the Economics of Covid-19. IZA Discussion Paper 2020, 13411. https://ssrn.com/abstract=3636640.

[16] Janssen J., Bar-Yam Y. (2021). Lowest-Cost Virus Suppression. arXiv.

[17] López-Valcárcel B.G., Vallejo-Torres L. (2021). The costs of COVID-19 and the cost-effectiveness of testing. Appl. Econ. Anal..

[18] Eurostat Statistic Explained Online Publication Building the System of National Accounts—Basic Concepts. https://ec.europa.eu/eurostat/statistics-explained/index.php?title=Building_the_System_of_National_Accounts_-_basic_concepts.

[19] Harvey A.C. (1989). Structural Time Series and the Kalman Filter.

[20] Scott S.L., Varian H.R. (2014). Predicting the present with Bayesian Structural Time Series. Int. J. Math. Model. Numer. Optim..

[21] McQuire C., Tilling K., Hickman M., de Vocht F. (2019). Forecasting the 2021 local burden of population alcohol related harms using Bayesian structural time-series. Addiction.

[22] de Vocht F., Tilling K., Pliakas T., Angus C., Egan M., Brennan A., Cambell R., Hickman M. (2017). The intervention effect of local alcohol licensing policies on hospital admission and crime: A natural experiment using a novel Bayesian synthetic time-series method. J. Epidemiol. Community Health.

[23] Pinilla J., Negrín M., López-Valcárcel B.G., Vázquez-Polo F. (2018). Using a Bayesian Structural Time–Series Model to Infer the Causal Impact on Cigarette Sales of Partial and Total Bans on Public Smoking. Jahrbücher für Nationalökonomie und Statistik.

[24] Feroze N. (2020). Forecasting the patterns of COVID-19 and causal impacts of lockdown in top five affected countries using Bayesian Structural Time Series Models. Chaos Solitons Fractals.

[25] Brodersen K.H., Gallusser F., Koehler J., Remy N., Scott S.L. (2015). Inferring causal impact using Bayesian structural time-series models. Ann. Appl. Statatistics.

[26] CausalImpact B.K. (2015). An R Package for Causal Inference Using Bayesian Structural Time-Series Models. https://google.github.io/CausalImpact/CausalImpact.html.

[27] BBVA Research, Publicaciones de Análisis Regional España. https://www.bbvaresearch.com.

[28] Boscá J.E., Doménech R., Feri J. (2020). El Impacto Macroeconómico del Coronavirus. Fedea Apuntes.

[29] Fernández A. (2021). The economic performance of Spanish provinces during 2020 and its determinants. Banco de España. Econ. Bull. Anal. Artic..

[30] Conesa J.C., Fernández G., Kehoe T.J. (2020). La crisis económica de COVID-19: Vías de Salvación. Aspectos económicos de la crisis del Covid-19. Coordinadores Felgueroso, F., de la Fuente, A.; Jansen, M. Estudios sobre la Economía Española. FEDEA Boletín de Seguimiento.

[31] OECD Tourism Trends and Policies 2020. OECD Publishing, Paris. https://www.oecd.org/cfe/tourism/OECD-Tourism-Trends-Policies%202020-Highlights-ENG.pdf.

[32] Instituto Nacional de Estadística (INE), Movimientos turísticos en fronteras Frontur. https://www.ine.es/dyngs/INEbase/es/operacion.htm?c=Estadistica_C&cid=1254736176996&menu=ultiDatos&idp=1254735576863.

[33] Instituto Nacional de Estadística (INE), Índice de Producción Industrial Base 2015. https://www.ine.es/dyngs/INEbase/es/operacion.htm?c=Estadistica_C&cid=1254736145519&menu=resultados&idp=1254735576715#!tabs-1254736194962.

[34] Asociación Española de Fabricantes de Automóviles y Camiones (Anfac) Informe de Producción y Exportación de Vehículos. https://anfac.com/actualidad/la-produccion-de-espana-se-reduce-un-196-con-227-millones-de-vehiculos-fabricados-en-2020/.

[35] Ministerio de Trabajo y Economía Social Estad’sticas de Empresas Inscritas en la Seguridad Social. https://www.mites.gob.es/estadisticas/emp/welcome.htm.

[36] Instituto Nacional de Estadística (INE), Contabilidad Nacional Trimestral (CNTR). https://www.ine.es/dyngs/INEbase/es/operacion.htm?c=Estadistica_C&cid=1254736164439&menu=resultados&idp=1254735576581.

[37] Eurobarometer European Commission. https://ec.europa.eu/commfrontoffice/publicopinion/index.cfm.

